# Confirming the TMEM232 gene associated with atopic dermatitis through targeted capture sequencing

**DOI:** 10.1038/s41598-021-01194-6

**Published:** 2021-11-08

**Authors:** Jie Zheng, Yuan-yuan Wu, Wen-liang Fang, Xin-ying Cai, Zeng-yun-ou Zhang, Chong-xian Yu, Xiao-dong Zheng, Feng-li Xiao

**Affiliations:** 1 Department of Dermatology of First Affiliated Hospital, and Institute of Dermatology, Anhui Medical University, Science and Teaching Building, 81 Meishan Road, Hefei City, 230032 Anhui Province China; 2 Key Laboratory of Dermatology, Anhui Medical University, Ministry of Education, China, Hefei, Anhui China; 3 Clinical College, Anhui Medical University, Hefei, Anhui China; 4 The Center for Scientific Research of Anhui Medical University, Hefei, Anhui China

**Keywords:** Computational biology and bioinformatics, Genetics, Immunology

## Abstract

Atopic dermatitis (AD) is a common and complex skin disorder, and the 5q22.1 region had been reported to be associated with AD. To confirm the susceptibility gene for AD in the 5q22.1 region by haplotype and targeted capture sequencing. The haplotypes were reconstructed with the genotyping data of four SNPs and six deletions from 3624 Chinese Hans AD patients and 5076 controls. The targeted capture sequencing spanning 5q22.1 region was performed in the selected samples. The gene level enrichment analysis was done using loss of function variants. A total of 62 haplotypes were found, and the H15 haplotype had the strongest association with AD (*P* = 3.92 × 10^−10^, OR 0.17, 95% CI 0.09–0.32). However, no co-segregation mutation sites were found in the sequencing analysis within the 16 selected samples, while the enrichment analysis indicated that TMEM232 was significantly associated with AD (P = 7.33 × 10^–5^, OR 0.33, 95% CI 0.19–0.58). This study confirms previous findings that the TMEM232 gene is associated with AD by haplotype analysis and targeted capture sequencing.

## Introduction

Atopic dermatitis (AD) is a common, multifactorial chronic inflammatory skin disease^1^. In recent years, the number of AD cases has gradually increased with a prevalence of 0.2–24.6% worldwide^2^. The aetiology of AD remains unclear. The dysregulation of immune response and defects in the epidermal barrier has a critical impaction in the development of AD, which was caused by the interactions between genetic and environmental factors^3,4^.

We have conducted a genome-wide association study (GWAS) of AD among the Chinese Han population in 2011, the previously undescribed susceptibility loci at 5q22.1(TMEM232 and SLC25A46) was identified to have an association with AD^5^, which was subsequently replicated in a Japanese AD GWAS^6^. To identify possible susceptibility genes in 5q22.1, we further scanned the indels and single-nucleotide polymorphisms (SNPs) in the 5q22.1 region through genomic imputation and genotyping. Six deletions and four SNPs were associated with AD. The strongest variant rs11357450 deletion is located in TMEM232. The protein expression of TMEM232 was different between AD and normal tissues by immunohistochemistry. TMEM232 may be a susceptibility gene for AD in the Chinese Han population^7^.

The haplotype may be more robust than the individual markers for delineating the susceptibility genes of complex disease^8^. The reconstructed haplotypes analysis were more reliable, cost-effective method to predict an individual’s reaction to a drug or risk of disease rather than single SNPs^9^. The haplotype studies of AD provided a molecular basis for explaining the pathogenesis of disease. Lacy et al^10^ found IgE levels in AD patients was associated with the haplotype TGAC in the IL10 promoter region. Tang et al^11^ performed genetic association study of the genotypes and haplotypes and implicated that SHARPIN may be a novel participant in the pathogenesis of AD. Our study intends to define haplotypes from the previous genotyping data in the 5q22.1 region^7^. High-throughput sequencing was used to discover whether there was a high-risk haplotype would co-exist with a gene function mutation, and loss of function (LOF) variants enrichment test in gene level will be performed to explore candidate genes for AD.

## Results

### The results of haplotype analysis

A total of 62 haplotypes were found in the discovery stage (Supplementary Table 1). The most frequent haplotype was H62, with a frequency of 23.19% in cases and 24.15% in controls (*P* = 1.56 × 10^−1^, OR 0.95, 95% CI 0.88–1.02). A significantly higher frequency of H1, H14, H30, H39, H43 was separately detected in patients than in controls (all *p* < 0.05). The frequencies of H6, H15, H47 and H53 were obviously lower in patients than in controls (all *p* < 0.05). And the most significant association was observed between H15 and AD (*P* = 3.92 × 10^−10^, OR 0.17, 95% CI 0.09–0.32) (Table 1).

**Table 1:** The common and significant haplotypes associated with atopic dermatitis in 5q22.1region.

Haplotype ID	Haplotype Genotype	Maf		*p* value	OR	95% CI
		Cases (%)	Controls (%)			
*H1	GCCTAACAGT	0.34	0.18	4.75 × 10–2	1.85	0.99–3.43
*H6	GCCTAGCGAT	0.1	0.45	7.72 × 10–5	0.23	0.1–0.51
*H14	**GCCTGGCGAT**	**6.8**	**5.52**	6.58 × 10–4	1.25	1.1–1.42
*H15	**GA CTG CA TAGACTAGTACA**	**0.16**	**0.94**	3.92 × 10–10	0.17	0.09–0.32
H27	GCTG CA TAAGCTAGTACA	0.55	0.47	4.94 × 10–1	1.16	0.75–1.79
*H30	GACCTGGCGAT	0.33	0.16	3.29 × 10–2	1.99	1.05–3.79
*H39	GA CTG CA TAAACAGT	0.56	0.25	1.07 × 10–3	2.3	1.38–3.83
*H43	**GA CTG CA TAAACTAGT**	**5.39**	**4.63**	2.49 × 10–2	1.18	1.02–1.36
*H47	**GA CTG CA TAAGCGAT**	**0.09**	**0.57**	5.46 × 10–7	0.15	0.07–0.36
*H53	GA CTG CA TAGACTAGT	0.01	0.09	4.68 × 10–2	0.16	0.02–1.27
**H54**	**GCCTGGCGATACA**	**0.55**	**0.56**	8.94 × 10–1	0.97	0.64–1.48
**H56**	**GA CTG CA TAGGCGAT**	**4.12**	**3.72**	1.94 × 10–1	1.11	0.95–1.3
**H62**	**GA CTG CA TAAACTAGTACA**	**23.19**	**24.15**	1.56 × 10–1	0.95	0.88–1.02

*Maf*, Minor Allele Frequency; The indels are marked with horizontal line. Bold font represent seven common haplotypes (> 0.5% freguency); *represent five haplotypes with statistically significant.

### The results of targeted next generation sequencing

Sixteen individuals (eight cases and eight controls) were selected for sequencing, which represented some common haplotypes (> 0.5% frequency). Eight cases included five with H15/H62 and three without H15 (H62/H23, H62/H23, H29/H62), eight controls consisted of one with H15 homozygous, three with H15 heterozygous (H15/H56, H14/H15, H15/H62) and four without H15 (H52/H62, H29/H62, H43/H56, H27/H54) (Supplementary Table 2).

We performed sequencing of 16 DNA samples with an average of 1629.37 Mb raw bases. After removing low-quality reads, we obtained an average of 10,760,164 clean reads (1602.37 Mb). The average GC content was 40.21%. The 1.79 Mb target region were captured, and 66.46% mapped to target regions of total effective bases. The mean sequencing depth on target regions was 499.02-fold.

The H15 was considered a research point to discover the causal mutation, but no co-segregated LOF variants with H15 were found in sequencing analysis, indicating that there is no gene mutation specific to H15. Since there were 11 genes in 5q22.1 regions included in the target sequence experiment, to explore whether these genes that have more than two LOF variants are associated with AD, we performed a chi-square test between case and control cohort by using LOF variants frequency that was calculated within one specific gene. The result indicated that TMEM232 was statistically significant associated with AD (*P* = 7.33 × 10^−5^, OR 0.33, 95% CI 0.19–0.58) and had the same direction as H15 (Table 2).

**Table 2:** The results of gene level loss of function variants enrichment test.

Gene	Cases		Controls		Chi-square	*P* value	OR	95% CI
	Alt (N)	Ref (N)	Alt (N)	Ref (N)				
TMEM232	20	172	50	142	15.72	7.33 × 10 − 5	0.33	0.19–0.58
CAMK4	23	73	24	72	0.03	8.67 × 10 − 1	0.95	0.49–1.83
EPB41L4A	155	149	157	147	0.03	8.71 × 10 − 1	1.00	0.71–1.34
LOC102467214	47	49	46	50	0.02	8.85 × 10 − 1	1.04	0.59–1.84
MAN2A1	118	90	118	90	0.00	/	1.00	0.68–1.47
SLC25A46	34	142	45	131	1.98	1.60 × 10 − 1	0.70	0.42–1.16
NREP	6	106	5	107	0.10	7.57 × 10 − 1	1.21	0.37–4.09
STARD4	4	28	7	25	0.99	3.20 × 10 − 1	0.51	0.13–1.95
TSLP	24	120	20	124	0.43	5.12 × 10 − 1	1.24	0.65–2.36
WDR36	64	272	67	269	0.09	7.70 × 10 − 1	0.95	0.65–1.38
LOC100289673	0	32	2	30	2.07	1.51 × 10 − 1	1.07	0.98–1.17

*Alt* alternative allele; Ref, the allele in the reference genome. N, LOF variants counts in case and control cohort that were separated by with or without H15 haplotype.

## Discussion

The GWAS studies have identified 147 risk loci for AD (search in GWAS catalog website), these loci also suggest that genes in immune responses and epidermal skin barrier functions are associated with AD^12–15^. In fact, the overall study of different SNP sites is more conductive for discovering genes associated with a disease or a certain phenotype^16^. Theoretically, the SNPs in proximity should exhibit high LD^17^, Many studies based on haplotypes can bring us more efficiency than a single SNP study^18,19^.

The haplotypes spanning the 5q22.1 region were constructed by the genotypes of 10 variants from our previous AD studies^7^^.^ We added 612 samples and performed on 3624 patients (2178 men and 1446 women), with a mean age of 5.75 ± 7.82 years old, and 5076 controls (2511 men and 2565 women) with a mean age of 28.4 ± 13.6 years old. The frequency of H15 haplotype was the most significantly different between AD patients and controls, so it was considered a research point to discover the causal mutation. By targeted resequencing of risk and non-risk associated haplotypes in the certain locus, the functional risk variants may be identied^20^. However, the target sequence experiment failed to identify causal variant that was co-segregated with H15 haplotype in this study.

The LOF variants of certain gene, including of exonic, UTR, splicing site or upstream, have been inconsistently associated with altered gene expression and/or directly with disease^21^. In order to find the susceptibility genes associated with AD, the data of target sequence at 5q22.1 region was used to make gene level enrichment analysis. The number of LOF variants at TMEM232 gene was significantly associated with AD. It is consistent with our previous result by the fine gene mapping^7^. Meanwhile, it was interesting that the associated direction of this gene is exactly the same as H15 haplotype, which indicate there maybe have a linkage between this gene and haplotype H15 while in a large sample size.

TMEM232 belongs to the transmembrane protein family (TMEMs), including TMEM45A, TMEM45B, and TMEM79 and et al., which have been predicted to be components of cellular membranes, such as mitochondrial membranes, the endoplasmic reticulum, lysosomes and the Golgiapparatus^22^. A recent study identified nonsense and missense mutations of the TMEM79 gene that encode the protein mattrin in some Irish AD patients who lack an FLG mutation^23^. TMEM45A is associated with the Golgi apparatus, with the trans-Golgi/trans-Golgi network in vitro and in the granular layer in vivo, which shows a strong correlation between TMEM45A expression and epidermal keratinization^24^. There are few reports about the TMEM232 gene, and its function is not clear. We analysed the expression of TMEM232 gene in HaCaT cells by western blots. Comparing with the HaCat cells control group, the level of Th2 cytokines IL4 and IL13 increased in TMEM232 gene overexpression HaCat cells group, and decreased in TMEM232 gene silence HaCat cells group. Further mechanistic studies will also be required to confirm that TMEM232 is causal for AD.

Our previously study indicated that one indel SNP rs11357450 that located in TMEM232 is associated with AD^7^. In this present study, we further investigated the haplotypes in 5q22.1 region with ten SNPs, and found one haplotype was significantly associated with AD. And with target sequence to these samples that have H15 haplotype, this haplotype was found also linked with functional mutations located within TMEM232 gene. These new findings indicate TMEM232 maybe the pathogenic gene for the progress of AD and further confirm the speculation of previous study.

There were limitations in this study. Although the target sequence experiment covered 2.9 Mb region, it didn’t overlap the entire 5q22 region. Further research on larger sample sizes, including more high frequency and significant haplotypes, is needed to search for functional gene mutations. In addition, more gene function exploration and the possible underlying mechanisms still require further research.

We confirm previous findings that the TMEM232 gene is associated with AD by haplotype analysis and targeted capture sequencing.

## Methods

### Study samples

This study was performed on 3624 patients (2178 men and 1446 women), with a mean age of 5.75 ± 7.82 years old, and 5076 controls (2511 men and 2565 women) with a mean age of 28.4 ± 13.6 years old. All participants were unrelated and of Chinese Han origin. The clinical information was collected through comprehensive clinical examinations. The diagnosis of AD was made by at least two experienced dermatologists based on the standard criteria of Hanifin and Rajka criteria^25^. All control groups were healthy individuals without AD, other atopic diseases, systemic diseases, or a family history of AD (including first-, second- and third-degree relatives). All participants or their guardians received written informed consent. The study was conducted in accordance with the Declaration of Helsinki principles and was approved by the Institutional Ethics Committee of Anhui Medical University.

### Defining haplotypes

The genotyping data of the significant four SNPs (rs10067777, rs7701890, rs13360927 and rs13361382) and six deletions (rs5870408, rs140764268, s11357450, rs35639206, rs137936676, rs10617471) were extracted spanning the 5q22.1 region from our previous AD studies^5,7^. These variants were in strong linkage disequilibrium (LD: r2 ≥ 0.80). The PHASE v 2.1 was used for reconstructing haplotypes from our AD genotype data^26,27^. Then, the local Perl script was applied to convert the polymorphic results of haplotypes into biallelic format, and the p-value and corresponding odds ratio (OR) with a 95% confidence interval were computed using Chi-square tests implemented in PLINK version1.07.

### Selecting samples for sequencing

Some samples were selected for sequencing based on the haplotype association results and the quality of remainder DNA samples that fully meet the high-throughput sequencing requirements. Firstly, the samples with the top significant haplotypes were prioritized. Secondly, one with this homozygote haplotype was screened as many as possible, and that with heterozygotes haplotype were selected when a sufficient number of homozygotes were not available. Third, the selected samples represented each high frequency haplotype (> 0.5%). At last, two samples without the haplotype were screened as a reference.

### Targeted next generation sequencing

The genomic DNA samples that met the criteria were randomly fragmented by Covaris technology. The size of the library fragments was mainly distributed between 150 and 250 bp. The end of DNA fragments was repaired and an "A" base was added at the 3'-end of each strand. Adaptors were then connected to both ends of the end repaired/dA tailed DNA fragments for amplification and sequencing. Selected DNA fragments were amplified by ligation-mediated PCR (LM-PCR), purified, and hybridized to the targeted region array for enrichment. Non-hybridized fragments were then removed by washing. Captured products were then circularized. DNA nanoballs (DNBs) were produced by rolling circle amplification (RCA). Each qualified captured library was then loaded on the BGISEQ-500 sequencing platform and high-throughput sequencing is performed on each capture library to ensure that each sample met the required average sequencing coverage. The targets covered approximately 2.9 Mb (from 109 to 112 M) of the 5q22.1 region and its adjacent sequences.

### Mapping and variants calling

To reduce the noise of sequencing data, data filtering was carried out as follows: removing reads containing sequencing adaptors; removing reads whose low-quality base ratio (base quality less than or equal to 5) is more than 50%; and removing reads whose unknown base ('N' base) ratio is more than 10%. Statistical analysis of data and downstream bioinformatics analysis were performed on this filtered, high-quality data, referred to as the "clean data".

All clean data of each sample were mapped to the human reference genome (GRCh37/HG19). The alignment was performed by Burrows-Wheeler Aligner (BWA) software. To ensure accurate variant calling, we followed the recommended Best Practices for variant analysis with the Genome Analysis Toolkit (GATK, https://www.broadinstitute.org/gatk/guide/best-practices). GATK was used to recalibrate base quality score and realign local around indels, with duplicate reads removed by Picard tools. HaplotypeCaller of GATK was used to detected genomic variations, including SNPs and indels. The variant quality score recalibration (VQSR) method, which uses machine learning to identify annotation profiles of variants that are likely to be real, was used to obtain high-confident variant calls, and all variant calls were annotated by ANNOVER.

### Gene level enrichment analysis

Variants annotated by ANNOVA as exonic, UTR, splicing site or upstream will consider to be LOF variants. All these variants were detected manually to find out whether they were co-segregation with the most significant haplotype. With an in-house perl script to count LOF variants number in case and control cohorts respectively, the gene level enrichment analysis was performed using chi-square test on genes that had two or more LOF variants to detect the susceptibility genes of AD.

## Supplementary Information

Supplementary Information.

## References

[1] Bieber T (2008). Atopic dermatitis. N. Engl. J. Med..

[2] Odhiambo JA, Williams HC, Clayton TO, Robertson CF, Asher MI (2009). Global variations in prevalence of eczema symptoms in children from ISAAC Phase Three. J. Allergy Clin. Immunol..

[3] Peng W, Novak N (2014). Recent developments in atopic dermatitis. Curr. Opin. Allergy Clin. Immunol..

[4] Vuyst ED, Salmon M, Evrard C, Rouvroit CLD, Poumay Y (2017). Atopic dermatitis studies through in vitro models. Front. Med..

[5] Sun LD (2011). Genome-wide association study identifies two new susceptibility loci for atopic dermatitis in the Chinese Han population. Nat. Genet..

[6] Hirota T (2012). Genome-wide association study identifies eight new susceptibility loci for atopic dermatitis in the Japanese population. Nat. Genet..

[7] Wu YY (2017). Scanning indels in the 5q22.1 region and identification of the TMEM232 susceptibility gene that is associated with atopic dermatitis in the Chinese Han population. Gene.

[8] Akey J, Jin L, Xiong M (2001). Haplotypes vs single marker linkage disequilibrium tests: What do we gain?. Eur. J. Human Genet. Ejhg.

[9] Davidson S (2000). Research suggests importance of haplotypes over SNPs. Nat. Biotechnol..

[10] Lacy K, Archer C, Wood N, Bidwell J (2010). Association between a common IL10 distal promoter haplotype and IgE production in individuals with atopic dermatitis. Int. J. Immunogenet..

[11] Tang L, Wang J, Zhu J, Liang Y (2018). Down-regulated SHARPIN may accelerate the development of atopic dermatitis through activating interleukin-33/ST2 signalling. Exp. Dermatol..

[12] Bin L, Leung DY (2016). Genetic and epigenetic studies of atopic dermatitis. Allergy Asthma Clin. Immunol..

[13] Ishigaki K (2020). Large-scale genome-wide association study in a Japanese population identifies novel susceptibility loci across different diseases. Nat. Genet..

[14] Marenholz I (2015). Meta-analysis identifies seven susceptibility loci involved in the atopic march. Nat. Commun..

[15] Paternoster L (2015). Multi-ancestry genome-wide association study of 21,000 cases and 95,000 controls identifies new risk loci for atopic dermatitis. Nat. Genet..

[16] Soyama A (2006). Sequence-based analysis of the CYP2D6*36-CYP2D6*10 tandem-type arrangement, a major CYP2D6*10 haplotype in the Japanese population. Drug Metab. Pharmacokinet..

[17] Altshuler D, Donnelly P (2005). A haplotype map of the human genome. Nature.

[18] Clark AG (2004). The role of haplotypes in candidate gene studies. Genet. Epidemiol..

[19] Schaid DJ (2004). Evaluating associations of haplotypes with traits. Genet. Epidemiol..

[20] Grassmann F (2015). A Candidate gene association study identifies DAPL1 as a female-specific susceptibility locus for age-related macular degeneration (AMD). Neuromol. Med..

[21] Monson ET (2016). Whole-gene sequencing investigation of SAT1 in attempted suicide. Am. J. Med. Genet. B Neuropsychiatr. Genet..

[22] Wrzesiński T (2015). Expression of pre-selected TMEMs with predicted ER localization as potential classifiers of ccRCC tumors. BMC Cancer.

[23] Saunders SP (2013). Tmem79/Matt is the matted mouse gene and is a predisposing gene for atopic dermatitis in human subjects. J. Allergy Clin. Immunol..

[24] Hayez A (2014). High TMEM45A expression is correlated to epidermal keratinization. Exp. Dermatol..

[25] Hanifin J (1980). Diagnostic features of atopic dermatitis. Acta Derm Venereol..

[26] Stephens M, Scheet P (2005). Accounting for decay of linkage disequilibrium in haplotype inference and missing-data imputation. Am. J. Hum. Genet..

[27] Stephens M, Smith NJ, Donnelly P (2001). A new statistical method for haplotype reconstruction from population data. Am. J. Hum. Genet..

