# Supplementary material for: Confirming the TMEM232 gene associated with atopic dermatitis through targeted capture sequencing
Source: Sci Rep. 2021 Nov 8;11:21830. doi: 10.1038/s41598-021-01194-6 (PMC8576034; doi:10.1038/s41598-021-01194-6)
Supplement: Supplementary file 1 — Supplementary Information. [file 41598_2021_1194_MOESM1_ESM.pdf]

Supplementary table 1 The common and significant haplotypes associated with atopic dermatitis in 5q22.1region

Haplotype ID	Haplotype Genotype	Maf		*p* value	OR	95% CI		
		Cases (%)	Controls(%)					
H1	GCCTAACAGT	0.34	0.18	4.75×10-2	1.85	1.00	~	3.43
H2	GCCTAACAG*TACA*	0.04	0.01	1.65×10-1	4.34	0.45	~	41.70
H3	GCCTAACGAT	0.07	0.03	2.14×10-1	2.41	0.58	~	10.09
H4	GCCTAA*CT*AGT	0.03	0.01	3.64×10-1	2.89	0.26	~	31.89
H5	GCCTAA*CT*AG*TACA*	0.13	0.18	4.24×10-1	0.72	0.32	~	1.61
H6	GCCTAGCGAT	0.10	0.45	7.72×10-5	0.23	0.10	~	0.51
H7	GCCTAGCGA*TACA*	0.01	0.01	7.93×10-1	1.45	0.09	~	23.11
H8	GCCTAG*CT*GAT	0.02	0.04	7.06×10-1	0.72	0.13	~	3.95
H9	GCCTGACGAT	0.01	0.01	7.93×10-1	1.45	0.09	~	23.11
H10	GCCTGA*CT*AG*TACA*	0.01	0.01	7.93×10-1	1.45	0.09	~	23.11
H11	GCCTGGCAAT	0.02	0.01	3.64×10-1	2.89	0.26	~	31.89
H12	GCCTGGCAGT	0.01	0.06	1.52×10-1	0.24	0.03	~	2
H13	GCCTGGCAG*TACA*	0	0.04	9.61×10-2	0	0	~	nan
H14	GCCTGGCGAT	6.80	5.52	6.58×10-4	1.25	1.10	~	1.42
H15	*GA* *CTG* *CA* *TA*GA*CT*AG*TACA*	0.16	0.94	3.92×10-10	0.17	0.09	~	0.32
H16	GCCTGGCGGT	0	0.03	1.50×10-1	0	0	~	nan
H17	GCC*TA*AA*CT*AG*TACA*	0.01	0.01	7.93×10-1	1.45	0.09	~	23.11
H18	GC*CA*TGGCGAT	0.01	0.01	7.93×10-1	1.45	0.09	~	23.11
H19	GC*CA* *TA*AA*CT*AG*TACA*	0.01	0.02	7.90×10-1	0.72	0.07	~	7.97
H20	G*CTG*CTGGCGAT	0.07	0.02	1.00×10-1	3.61	0.70	~	18.64
H21	G*CTG*C*TA*AA*CT*AG*TACA*	0	0.03	1.50×10-1	0	0	~	nan

H22	G*CTG*C*TA*AG*CT*AG*TACA*	0.01	0.02	7.89×10-1	0.72	0.07	~	7.97
H23	G*CTG*C*TA*GA*CT*AG*TACA*	0.03	0	8.91×10-2	NA	NA	~	NA
H24	G*CTG* *CA*TGGCGAT	0	0.02	2.39×10-1	0	0	~	nan
H25	G*CTG* *CA* *TA*AA*CT*AGT	0.09	0.08	8.81×10-1	1.08	0.37	~	3.12
H26	G*CTG* *CA* *TA*AA*CT*AG*TACA*	0.40	0.23	5.91×10-2	1.70	0.97	~	2.97
H27	G*CTG* *CA* *TA*AG*CT*AG*TACA*	0.55	0.47	4.94×10-1	1.16	0.75	~	1.79
H28	G*CTG* *CA* *TA*GA*CT*AG*TACA*	0.01	0.01	7.93×10-1	1.45	0.09	~	23.11
H29	G*CTG* *CA* *TA*GG*CT*AG*TACA*	0	0.02	2.39×10-1	0	0	~	nan
H30	*GA*CCTGGCGAT	0.33	0.16	3.29×10-2	1.99	1.05	~	3.79
H31	*GA*CCTGGCGA*TACA*	0.07	0.13	2.57×10-1	0.56	0.20	~	1.56
H32	*GA*C*CA* *TA*GG*CT*GA*TACA*	0.03	0	8.91×10-2	NA	NA	~	NA
H33	*GA* *CTG*CTAA*CT*AG*TACA*	0.01	0.02	7.90×10-1	0.72	0.06	~	7.97
H34	*GA* *CTG*CTGGCAG*TACA*	0.03	0.05	5.07×10-1	0.58	0.11	~	2.98
H35	*GA* *CTG*CTGGCGAT	0	0.03	1.50×10-1	0	0	~	nan
H36	*GA* *CTG*C*TA*AACGAT	0	0.02	2.39×10-1	0	0	~	nan
H37	*GA* *CTG*C*TA*GA*CT*AG*TACA*	0.01	0.04	3.42×10-1	0.36	0.04	~	3.23
H38	*GA* *CTG* *CA*TAA*CT*AG*TACA*	0.01	0.03	7.90×10-1	0.72	0.07	~	7.97
H39	*GA* *CTG* *CA* *TA*AACAGT	0.56	0.25	1.07×10-3	2.30	1.38	~	3.83
H40	*GA* *CTG* *CA* *TA*AACGAT	0.74	0.78	7.81×10-1	0.95	0.66	~	1.36
H41	*GA* *CTG* *CA* *TA*AACGA*TACA*	0.01	0.05	2.27×10-1	0.29	0.03	~	2.47
H42	*GA* *CTG* *CA* *TA*AA*CT*AA*TACA*	0	0.05	6.28×10-2	0	0	~	nan
H43	*GA* *CTG* *CA* *TA*AA*CT*AGT	5.39	4.63	2.49×10-2	1.18	1.02	~	1.36
H44	*GA* *CTG* *CA* *TA*AA*CT*GAT	0.04	0.07	4.83×10-1	0.62	0.16	~	2.40
H45	*GA* *CTG* *CA* *TA*AA*CT*GA*TACA*	0.06	0.11	2.62×10-1	0.53	0.17	~	1.65
H46	*GA* *CTG* *CA* *TA*AA*CT*GG*TACA*	0.04	0.12	9.96×10-2	0.36	0.10	~	1.28
H47	*GA* *CTG* *CA* *TA*AGCGAT	0.09	0.57	5.46×10-7	0.15	0.07	~	0.36

H48	*GA* *CTG* *CA* *TA*AGCGA*TACA*	0.04	0.02	3.85×10-1	2.17	0.36	~	12.98
H49	*GA* *CTG* *CA* *TA*AG*CT*AG*TACA*	0.31	0.39	4.07×10-1	0.80	0.47	~	1.36
H50	*GA* *CTG* *CA* *TA*AG*CT*GG*TACA*	0	0.02	2.40×10-1	0	0	~	nan
H51	*GA* *CTG* *CA* *TA*GACAG*TACA*	0	0.02	2.40×10-1	0	0	~	nan
H52	*GA* *CTG* *CA* *TA*GACGAT	0.01	0.02	7.90×10-1	0.72	0.07	~	7.97
H53	*GA* *CTG* *CA* *TA*GA*CT*AGT	0.01	0.09	4.68×10-2	0.16	0.02	~	1.27
H54	GCCTGGCGA*TACA*	0.55	0.56	8.94×10-1	0.97	0.64	~	1.48
H55	*GA* *CTG* *CA* *TA*GGCAG*TACA*	0.01	0.03	5.18×10-1	0.48	0.05	~	4.63
H56	*GA* *CTG* *CA* *TA*GGCGAT	4.12	3.72	1.94×10-1	1.11	0.95	~	1.30
H57	*GA* *CTG* *CA* *TA*GGCGA*TACA*	0.28	0.21	3.20×10-1	1.37	0.73	~	2.58
H58	*GA* *CTG* *CA* *TA*GGCGGT	0.01	0.02	7.90×10-1	0.72	0.07	~	7.97
H59	*GA* *CTG* *CA* *TA*GG*CT*AG*TACA*	0.07	0.05	5.58×10-1	1.45	0.42	~	4.99
H60	*GA* *CTG* *CA* *TA*GG*CT*GAT	0.04	0.03	6.50×10-1	1.45	0.29	~	7.16
H61	*GA* *CTG* *CA* *TA*GG*CT*GA*TACA*	0.39	0.27	1.81×10-1	1.45	0.84	~	2.49
H62	*GA* *CTG* *CA* *TA*AA*CT*AG*TACA*	23.19	24.15	1.56×10-1	0.95	0.88	~	1.02

Maf, Minor Allele Frequency. The indels are marked with horizontal line.

Supplementary Table 2 Sixteen individuals were selected for sequencing

Sample	Case/Control	Genotype	Symbol	Note
1	Case	H15/H62	0/1	
2	Case	H15/H62	0/1	
3	Case	H15/H62	0/1	
4	Case	H15/H62	0/1	
5	Case	H15/H62	0/1	
6	Case	H23/H62	0/0	without H15, H23 < 0.5%
7	Case	H23/H62	0/0	without H15, H23 < 0.5%
8	Case	H37/H62	0/0	without H15, H37 < 0.5%
9	Control	H15/H56	0/1	
10	Control	H14/H15	0/1	
11	Control	H15/H62	0/1	
12	Control	H15/H15	1/1	
13	Control	H52/H62	0/0	without H15, H52 < 0.5%
14	Control	H29/H62	0/0	without H15, H29 < 0.5%
15	Control	H43/H56	0/0	without H15
16	Control	H27/H54	0/0	without H15
